# Probabilistic base calling of Solexa sequencing data

**DOI:** 10.1186/1471-2105-9-431

**Published:** 2008-10-13

**Authors:** Jacques Rougemont, Arnaud Amzallag, Christian Iseli, Laurent Farinelli, Ioannis Xenarios, Felix Naef

**Affiliations:** 1 School of Life Sciences, Ecole Polytechnique Fédérale de Lausanne (EPFL), 1015 Lausanne, Switzerland; 2 Ludwig Institute for Cancer Research (LICR), Bâtiment Génopode, Université de Lausanne, 1015 Lausanne, Switzerland; 3 Swiss Institute of Bioinformatics (SIB), Bâtiment Génopode, Université de Lausanne, 1015 Lausanne, Switzerland; 4 Vital-IT, Bâtiment Génopode, Université de Lausanne, 1015 Lausanne, Switzerland; 5 Fasteris SA, P.O. box 28, 1228 Plan-les-Ouates, Switzerland

## Abstract

**Background:**

Solexa/Illumina short-read ultra-high throughput DNA sequencing technology produces millions of short tags (up to 36 bases) by parallel sequencing-by-synthesis of DNA colonies. The processing and statistical analysis of such high-throughput data poses new challenges; currently a fair proportion of the tags are routinely discarded due to an inability to match them to a reference sequence, thereby reducing the effective throughput of the technology.

**Results:**

We propose a novel base calling algorithm using model-based clustering and probability theory to identify ambiguous bases and code them with IUPAC symbols. We also select optimal sub-tags using a score based on information content to remove uncertain bases towards the ends of the reads.

**Conclusion:**

We show that the method improves genome coverage and number of usable tags as compared with Solexa's data processing pipeline by an average of 15%. An R package is provided which allows fast and accurate base calling of Solexa's fluorescence intensity files and the production of informative diagnostic plots.

## Background

Ultra-high-throughput sequencing is having a growing impact on biological research by providing a fast and high resolution access to genome-scale information. The versatile technique can be used for unbiased genotyping [1-3], transcriptome analysis [4-6], protein-DNA interactions[7,8], *de-novo *sequencing[9,10]. While the sample processing is relatively streamlined, innovations in data management and information processing are necessary to exploit the full potential of the technology. A standard Solexa/Illumina Genome Analyzer "classic" run produces 700 Gb of image files and 200 Gb of processed data files over 3.5 days totaling nearly 400,000 image files and 20,000 processed files. The latest GAII upgrade further increases this volume of data, mostly by acquiring larger images (although only 100 tiles) and with the ability to perform paired-end sequencing (72 bases per colony). The computing infrastructure required for managing daily sequencing runs is extremely costly to set up and maintain. Developing new algorithms to extract more information from available images and reduce the number of sequencing runs per project will therefore prove extremely valuable. Finally, well-designed quality metrics and diagnostic tools will allow a rapid assessment of the quality of the sequencing runs and decide the applicable data retention policy.

The Solexa/Illumina Genome Analyzer performs sequencing-by-synthesis of a random array of clonal DNA colonies attached to the surface of a flow cell. There are about 8 million such colonies on each of the 8 lanes of the cell. At each cycle of synthesis all four nucleotides, labelled with four different fluorescent dyes and blocked at the 3'-ends, are introduced in the flow cell. Up to 36 such cycles of synthesis are performed.

The data acquisition on the Genome Analyzer "classic" proceeds as follows: each lane of the cell is divided into roughly 300 tiles that are individually photographed through four different filters. The image analysis software localizes each colony on each picture and quantifies the corresponding four fluorescence intensities. The output consists of one file per tile with one row per colony made of four coordinates and up to 144 real numbers for 36 intensity quadruples. The base calling starts downstream of this quantification and reconstructs the DNA sequence that likely generated each colony. The Solexa data analysis pipeline outputs two important files for each tile in each lane: a sequence file with the sequence determined from each intensity row and a fast-q file with a quality score for each base called. This fast-q score measures the most likely base intensity relative to the three other intensities on a logarithmic scale from -5 to 40 (it is asymptotically equal to a Phred score[11]). Here we propose an alternative probabilistic base calling method based on the fluorescence intensity quantifications that uses the extended IUPAC alphabet to code ambiguous bases. An information criterion is used to control the length of trustable reads. We show that this methodology increases the specific mapping of the tags onto reference genomes by about 15% (typically 10–25%) on raw sequences and an increase of up to 70% after quality filtering. The method is implemented in a freely distributed software called Rolexa.

Similar approaches have recently been published. Closest to ours in their use of Gaussian mixtures is the method introduced by Cokus et al. in their analysis of Arabidopsis methylation patterns[12]. The Alta-Cyclic base caller [13] uses a support vector machine that needs to be trained on a known dataset. Our approach is computationally light and modular in that it offers a set of complementary functionalities that attempt to address the various biases observed in Solexa sequence [14-16] based on simple models of the biochemistry involved.

## Results

### Statistical properties of the fluorescent emissions

Several sources of noise perturb the acquisition step: signal over noise ratio in the images depends on the position of the colony within the imaging field (boundary effect), colonies can be hard to segment on the pictures, fluorophore emission spectra partially overlap as emissions "leak" into adjacent channels. Moreover synthesis efficiency is limited and therefore, within each colony, some DNA strands incorporate a non-complementary base or are de-synchronized because they failed to incorporate a nucleotide at a previous step. Both effects lead to the emission of a different fluorophore than the majority of the colony. These effects are possibly dependent on the base composition of the sequence[17] and are obviously deteriorating with each additional chemistry cycle.

**Figure 1:**
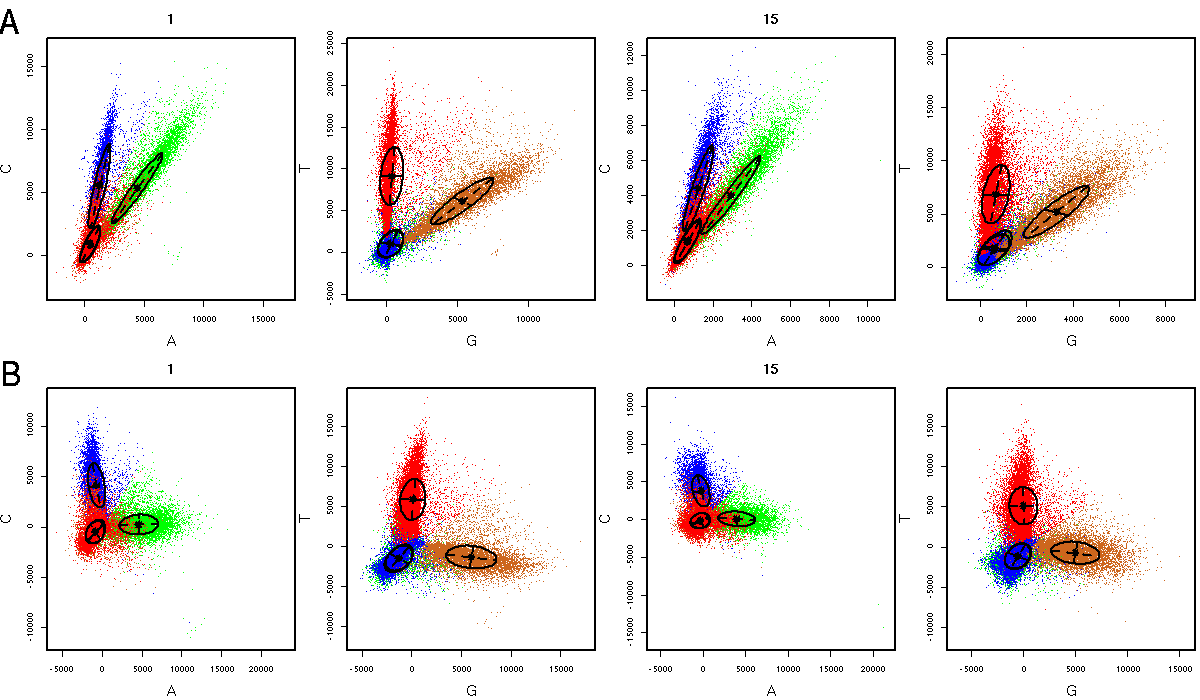
**Signal and noise in fluorescence intensities**. Representation of the first cycle of synthesis on five concatenated tiles of the phiX174 sequencing data. **A**. Projection of the intensity quadruples on the axes corresponding to the *A *and *C *channels and the *G *and *T *channels at cycles 1 an 15. The ellipses represent the Gaussian mixtures (centers and the line for one standard deviation are shown). **B**. Same data after de-correlation transformations (see Methods). Coloring reflects the mixture component with largest probability.

We use the sequencing of the phiX174 (see Material and Methods) to analyze the signal in the four color channels as the sequencing progresses. We first observe that the distribution of intensities in the individual channels shows a good separation between background noise and signal, although the shape of the histograms strongly depends on the dye used (Fig. 1A and Additional file 1). For example, *G *has a tighter dynamical range than *T *and the range generally decreases with the cycle number. The largest range spans 4–5 logs. As the sequencing progresses, dynamic range decreases, signal over noise ratios worsen and the separation between background noise and signal becomes increasingly blurred (Additional file 1). Next, we observe that the *A *and *C *channels, as well as the *T *and *G *channels, are highly correlated (Fig. 1A).

### Reducing positional bias, dephasing and cross-talk

As observed above, there are three main sources of systematic bias at the level of intensity data. The first is the cross-talk between color channels: for example the *A *and *C *channels are not independent. Thus we transformed the raw intensities by a linear mapping to the basis with axes at angles *ϕ *and *θ *with respect to the original axes (cf. methods). We optimize the two angles so as to minimize the overall correlation between the transformed coordinates. We repeat this operation at each cycle of sequencing as well as with the other two, *G *and *T *channels (Fig. 1B).

The second important bias is the colony dephasing: the amount of fluorescence emitted in a particular channel at cycle *n *depends on the number of corresponding bases present in the sequence at positions *1*, ..., *n*-*1 *because incorporation failures accumulated from previous cycles will be partly compensated at cycle *n *thereby increasing the signal in all channels. This cross-cycle dependence can be modelled by a binomial distribution with parameter *q *which is the probability of not elongating the complementary strand at each cycle of synthesis. We assume that this rate is equal for all nucleotides and all cycles. We determine the value of *q *by minimizing the average correlation between intensities at cycle *n *and *n+1*.

The last major source of systematic variation is due to an optical effect: on each tile, the colonies near the center of the image appear brighter than the ones near the edges (Additional file 2). We correct this by fitting a two-dimensional lowess [18] model to the intensities for each tile and subtracting the difference between the fit and the median intensity.

The three corrections are applied sequentially (cf. Methods) to the raw intensities before applying the model-based clustering algorithm described next.

### Model-based clustering and information-theoretic base calling

**Figure 2:**
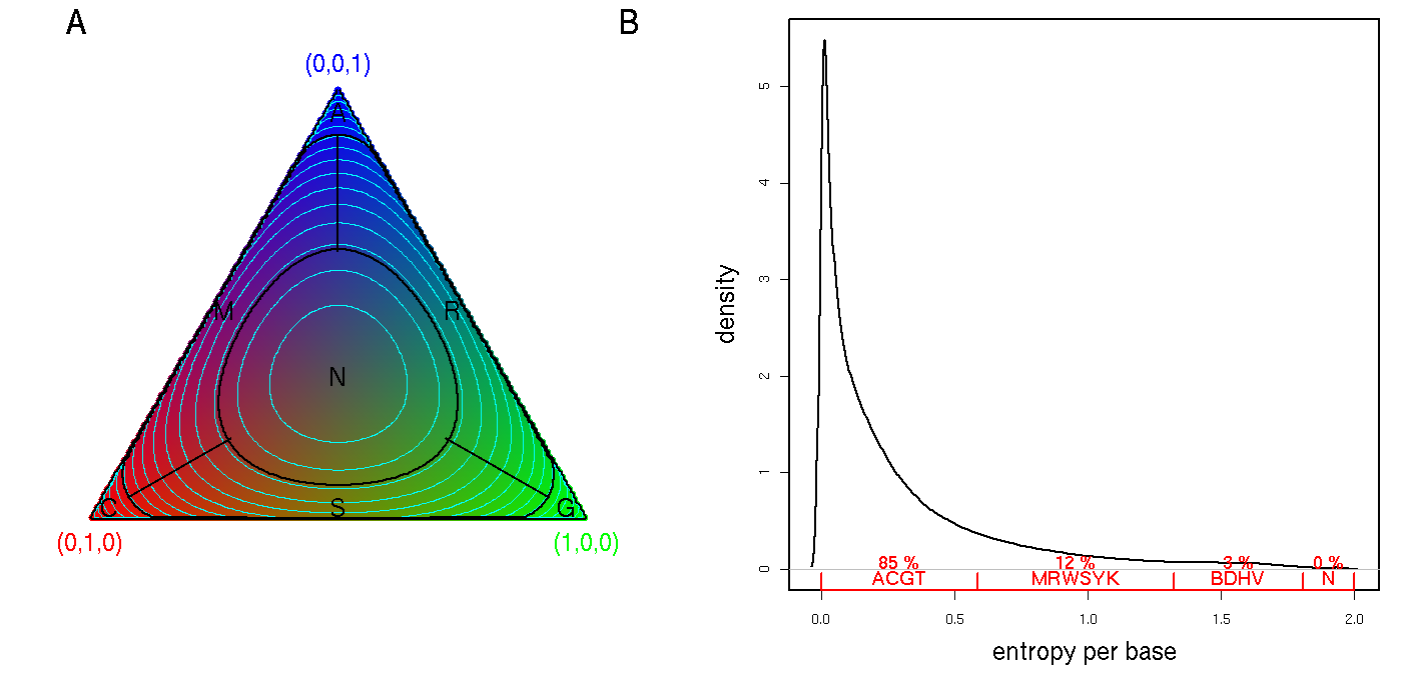
**Base calling determined by entropy**. **A**. Probability simplex for a 3-letter alphabet (*A *= blue, *C *= red, *G *= green). Each point in the triangle is a probability triplet (*P*_A_, *P*_C_, *P*_G_) represented by the corresponding color mixture. Blue lines are iso-entropic levels, black lines are the cutoffs between the various IUPAC codes. These correspond to midpoints in the state variable (*S *= 2^*h*^). **B**. Distribution of entropy per base across 10 tiles on 36 bases. Red lines at the bottom indicate the IUPAC cutoffs. Mass within each segment is indicated in red.

**Figure 3:**
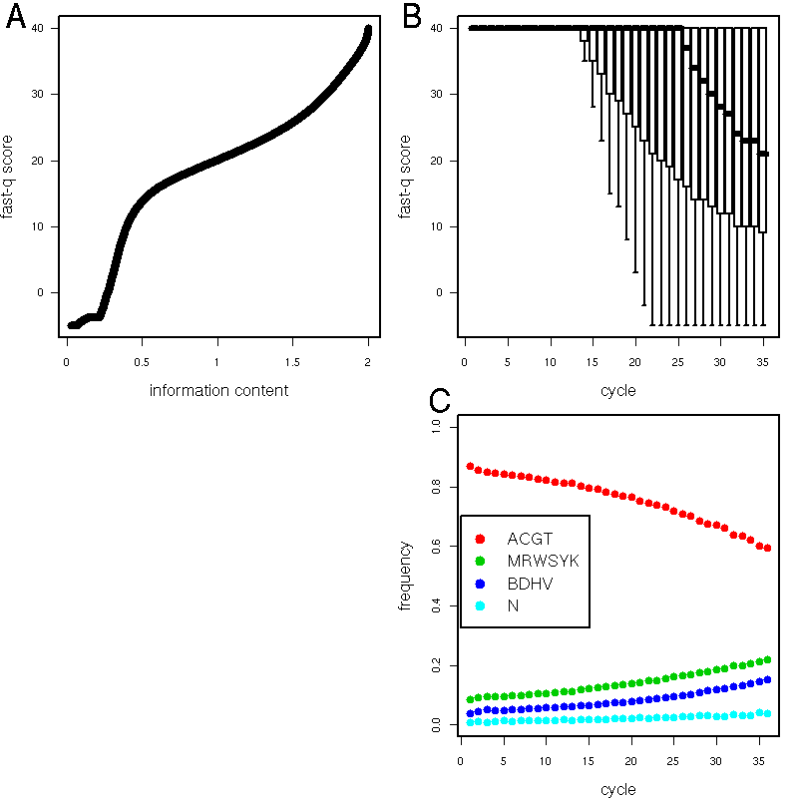
**Quality and entropy depend on position in the sequence**. **A**. Quantile-quantile plot of fast-q quality score against the information content per base. The two measures are loosely correlated, but clearly not equivalent. **B**. Boxplot of the fast-q score along the first 35 bases of the sequencing. The overall base quality decreases sharply after base 14, but the distribution still extends up to the top 40 score at bases 30–35. **C**. Frequency of the four categories of ambiguous IUPAC codes as a function of the position in the sequence.

We used a model-based clustering algorithm[12,19-22] to classify the intensity quadruples into four groups. Clearly, four well-delineated clusters corresponding to the four bases emerge (Fig. 1A–B). Specifically, we model the intensities measured in each channel by a mixture of four 4-dimensional Gaussian random variables and we use the intensity quadruples from all colonies in one or few combined tiles to fit the model parameters. The fitted model provides four probability distributions on the space of intensity quadruples, namely the probability *P*_A_(*k*) = *P*(*A*|*I*_1_(*k*), ..., *I*_4_(*k*)) that the *k*^th ^base to call is an *A *knowing the measured intensities in all four channels at cycle *k*, and similarly for *P*_C_, *P*_G _and *P*_T_. We can measure the level of uncertainty in our base calling by the entropy $h(k)=-\sum_{\alpha \in \{\text{ACGT}\}}P_{\alpha }(k)log_{2}P_{\alpha }(k)$ which measures the uncertainty (in bits) in the determination of the correct *k*^th ^base[23]. Knowing *h *and the four probabilities we then use cutoffs in the probability simplex to decide which IUPAC code to call (Figure 2A, Methods). As the sequencing progresses, we also compute the cumulative entropy of each colony, $H(n)=\sum_{k=1,\ldots ,n}h(k)$, which estimates the log_2 _of the number of actual sequences compatible with the codes called up to position *n*. This total entropy is used to rank tags from least to most ambiguous. Figure 3A shows that this ambiguity score correlates with, but is markedly different from the Solexa fast-q quality score. The ambiguity metric is useful for genome assembly or polymorphism identification by allowing down-weighting the low quality tags when deriving statistics from multiple alignments of tags. As shown below, this metric can also be used to optimize tag lengths and increase the chance of identifying a match on the reference genome.

### Genome coverage statistics

**Figure 4:**
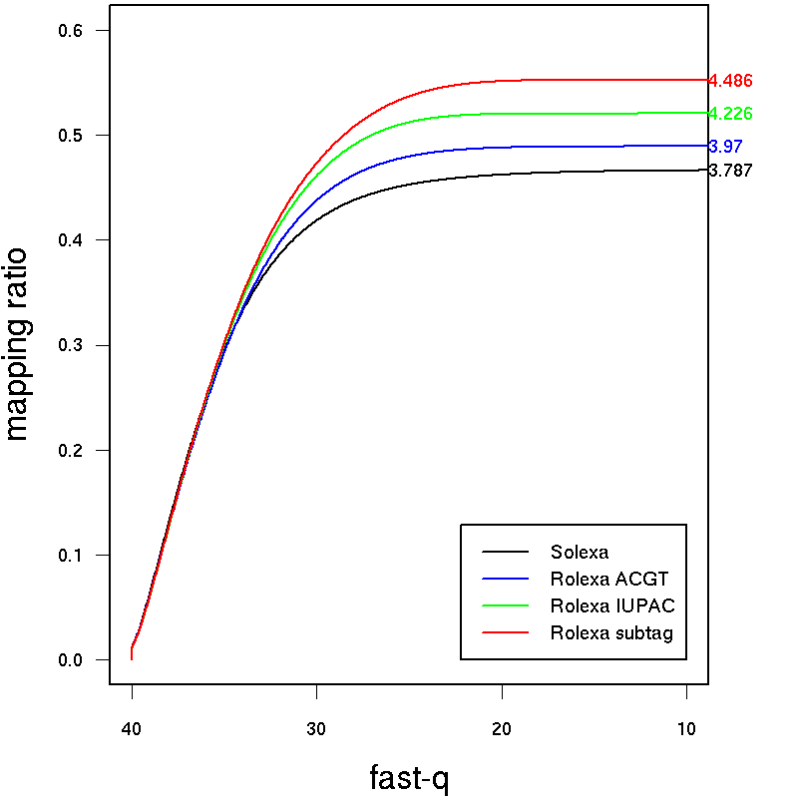
**Rolexa base-calling increases the coverage**. Black: Solexa base calling, blue: Rolexa base calling using only the ACGT alphabet (most probable base calling), green: Rolexa base calling using IUPAC codes, red: Rolexa base calling with IUPAC codes and tag length optimization. Numbers in the right margin are the number of matching tags in millions. Sequence tags were sorted by decreasing quality (fast-q) and unique exact matches on the reference phiX174 genome were searched. Vertical axis shows the proportion of tags finding an exact match.

To assess the quality of our base calling and to compare it with the sequences obtained via Solexa's analysis pipeline, we compute the mapping efficiency #{reads mapping exactly to the genome}/#{total number of reads}. We used the *fetchGWI *tool [24] to search for unique exact matches of each sequenced tag encoded in the IUPAC code on the 5386 nt reference phiX174 genome sequence [RefSeq:NC_001422]. We thus discard every tag that matches at more than one position or does not match exactly anywhere on the reference sequence. One lane (330 tiles) of the Solexa flow cell produced 8 M tags, 3 M unique tags and 3.8 mappable tags, which amounts to a throughput of 137 million immediately usable bases per run. Sorting tags by decreasing quality we see (Figure 4) that low-entropy tags are easily identified by both the Solexa and Rolexa pipelines, but that the coverage achieved by Rolexa-called tags increases significantly among the low-quality sequences and results in an increased total coverage of up to 10–25% (average 15%). We also see that ranking by quality (or entropy, data not shown) is a judicious prioritization strategy since the coverage increase is sharp in the top part of the list and subsequently plateaus off.

**Figure 5:**
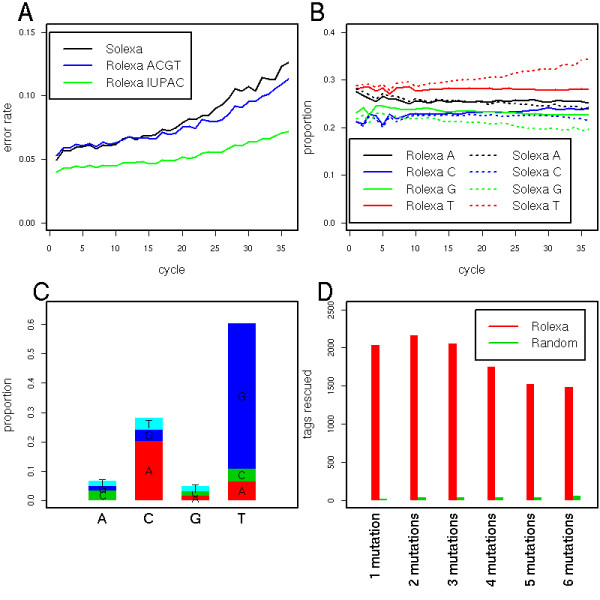
**Disequilibrium between complementary bases ratio**. **A**. Error rate at each cycle of sequencing. Each tag was aligned on the genome using *align0 *and the error rate defined by counting the number of differences between the bases called and the reference at the corresponding position. Black is the error rate for Solexa-called tags, blue for Rolexa tags called using only the ACGT alphabet and green for Rolexa-called tags with IUPAC codes. **B**. Proportion of bases *A*, *C*, *G *and *T *at each position in the tags for Solexa base calling (dashed lines) and Rolexa base calling (continuous line). The complementary *A *and *T *proportions are different (ratio is not 1) and are degrading along the sequences (lines drift apart). The proportions are less dependent on position with Rolexa base calling, although the ratios remain different from 1. Label on y-axis is wrong. Panels **C-D **focuses on tags "rescued" by Rolexa base calling, namely those tags that could not be mapped on the genome after Solexa base calling, but had a matching position via Rolexa base calling. **C**. The distribution of substitutions between the Solexa tags and the corresponding Rolexa tags shows a predominance of *C *to *A *and *T *to *G *substitutions which is consistent with a re-equilibration of the base complementarity.**D**. Introducing one to six mutations in the Solexa tags with the same frequencies as the Rolexa algorithm at random positions only rescues about 2% of the tags that were rescued by Rolexa with the same number of ambiguous bases (green bars).

To estimate error rates of sequencing, we used *align0 *[25] to search for an optimal match between each tag and the phiX genome, and then computed the number of mismatches between tag and reference. Figure 5A shows how the error rates increases as a function of the sequencing cycles for Solexa tags. Rolexa tags called with the most probable ACGT base showed a slower increase, and introducing IUPAC codes significantly decreased both the intercept and slope of the error rate as a function of the sequencing cycle.

### Base distribution statistics

A surprising property of Solexa sequences is the imbalance between complementary *A *and *T *base counts as well as between *G *and *C*[14]. As shown in Figure 5B, there is progressive deterioration in the proportions as the sequencing progresses, which is likely related to the varying noise levels across fluorescent dyes for complementary base pairs as well as dye-specific chemical effects (see Fig. 1). In consequence an intensity close to the background is more likely to be called *T *than *A*, or *C *than *G*. Applying our corrections at the level of intensities stabilizes the proportions of bases, which is particularly pronounced for the T's. For reasons we do not currently understand the A/T ratio is not exactly one but stabilizes around 0.9 (Figure 5B).

To ascertain whether our increased coverage is not simply the consequence of the more degenerate alphabet, we verified that introducing ambiguities at random positions does not similarly improve the mapping. We thus selected the tags that did not match on the genome based on Solexa base calling, but did match after Rolexa introduced one to five ambiguous bases. Then we introduced ambiguities in these tags, with the same frequency as Rolexa, but at random positions. Figure 5D shows that only about 2% of those randomized mutations found a match on the genome, indicating that the entropy is a specific predictor of ambiguous positions.

### Optimizing tag length

While Solexa's quality score tends to decrease along the sequence, its distribution mostly spreads, rather than shifts, downwards (Fig. 3B). Computing a global length cutoff based on the average quality will therefore discard a lot of high-quality bases and not necessarily ensure a uniform quality. Thus we expect to increase the number of tags that can be mapped to a reference sequence by cutting them to a shorter length [26]. However this procedure has a downside since it will reduce the coverage length per tag and increase the probability of finding multiple matches. Similarly, standard Solexa procedures suggest selecting tags with high average fast-q. Yet, a low average can be the result of just a few uncertain bases near the end of an otherwise useful tag.

**Figure 6:**
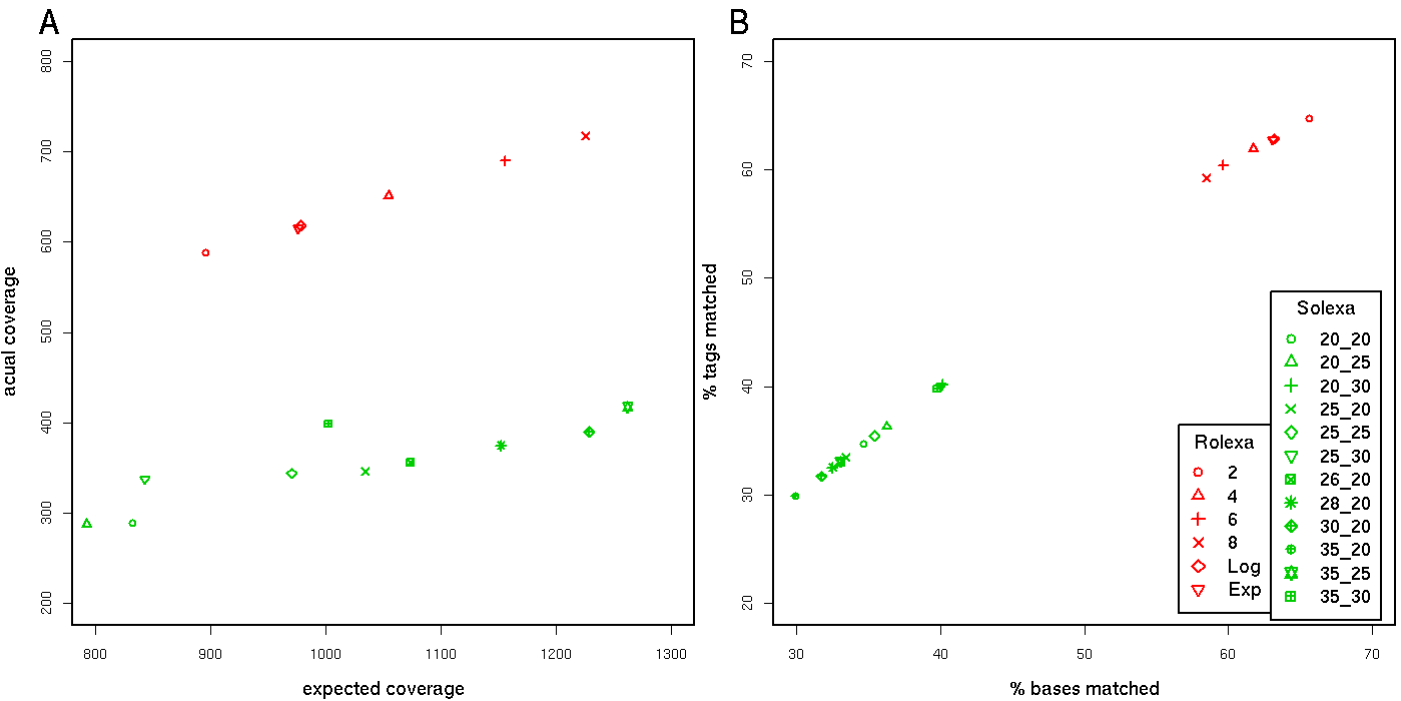
**Tag-dependent quality filtering improves the mapping efficiency**. Several entropy cutoffs were used to filter low-quality Rolexa-called tags and to reduce tags to higher scoring sub-tags. Solexa-called tags were filtered to the same length as the average length of the previous sets and to various average fast-q score. **A**. The actual coverage of the target genome as a function of the expected coverage (if all tags could have been mapped). **B**. The efficiency of the filtering in coverage ratio (actual number of nucleotides covered divided by expected number, X axis) and in tag mapping ratio (number of tags mapped to the genome divided by number of tags passing the quality filter, Y axis). Rolexa (red points) has superior efficiency to Solexa (green points) in all data sets. Points are labeled with the cutoffs used (see text): Rolexa cutoffs are either constant (2, 4, 6, 8), growing logarithmically (Log) or exponentially (Exp), Solexa cutoffs are indicated by two numbers, the length cutoff followed by the fast-q cutoff.

We tested the different selections by applying the following quality filters. For the Solexa method we cut the tags at length 20, 25, 26, 28, 30, and then filtered all sequences with average fast-q score bellow 30, 25, or 20. In comparison, we used the following filtering procedure for Rolexa tags: we chose 3 different length-dependent entropy cutoffs *IT(k) *(see methods) and searched within each read for the longest *k*-mer with total entropy less than *IT(k)*. We then extended this subsequence in both directions up to the next ambiguous base and eventually removed all tags shorter than 10 bases. The coverage statistics for the different filters are summarized in Figure 6. We performed a similar analysis of the 330 tiles of the sequencing of targeted human genomic regions and found an average of 50% increase in nucleotide coverage (Additional file 3). We see that the efficiency of Rolexa is superior in all datasets as measured by the ratio of actual coverage to expected coverage as well as by the ratio of tags having a unique match on the genome. The latter criterion is important since in many application of high-throughput sequencing (such as gene expression measures or ChIP-Seq), the extent of the coverage is less important than the number of hits on the genome. Similarly, in genotyping and targeted re-sequencing, where inexact matches are expected, the ability to reliably filter out low-quality tags before doing the matching to the reference sequence is of the highest importance, since actual polymorphisms must be distinguished from sequencing errors.

## Discussion

Several points in the analysis of Solexa high throughput sequencing technology can likely benefit from further improvements. First the disequilibrium between complementary bases should be reduced. Although the phiX174 is a single-stranded DNA virus, the library was prepared from the double-stranded covalently closed circular form of the genome. As shown, the output of the sequencing shows an increasing deterioration of the equilibrium between complementary bases as the sequencing cycles proceed (Figure 5B). Our approach improves on this but does not solve the issue completely.

Similar approaches have recently been Dohm et al.[14] have observed similar bias to the ones described here, but only proposed to correct them at the level of the sequence alignment, not at the level of the base calling. Cokus et al.[12] use Solexa's pre-treated data (_sig2 files) and apply a very similar EM procedure to fit a Gaussian mixture model for probabilistic base calling. They do not use information based metrics to reduce the probabilities to IUPAC codes, but rather construct position-weight matrices with which they scan the reference genome, which is computationally expensive and not directly applicable for *de-novo *sequencing. Erlich et al.[13] train a Support Vector Machine optimized on a reference sequence which is computationally highly expensive. Rolexa only needs a (nowadays common) multi-core computer and runs a complete analysis of one lane in 10 hours over 5 cores. Moreover it is based on modeling the bio-chemical properties of the system.

We have not considered here the potentially important benefits of fine-tuning the image analysis algorithms. Looking at images generated by the microscopic device shows that when the density of colonies is high in some region of the images, bleeding-over occurs and assigning the correct fluorescence intensity to each colony is clearly a delicate problem (see [16]).

Due to the large file size and format of the Solexa output data, concurrently (and randomly) accessing 20,000 text files puts a heavy strain on any standard file system, not to mention backup devices. Rolexa works with compressed inputs and outputs, which already reduces file size considerably. Still, a better suited file format could help both the storage and the processing, e.g. using suffix tables and trees[27,28]. The latest GAII upgrade to the Solexa/Illumina sequencer generates even more data, through larger acquisition area, longer reads, and paired-end sequencing. Generating longer reads require efficient and reliable algorithms for base calling with reasonable levels of accuracy up to the end of the read. Furthermore, this increased throughput requires these algorithms to be fast and be based on direct and simple methods that are re-usable without tuning from one run to the next.

## Conclusion

Solexa/Illumina high-throughput sequencing has already and will increasingly produce vast amounts of systems scale genomics and functional genomics data. As with other high-throughput techniques, improvements in signal processing and statistical assessment of the data will prove to be a key step in the maturation of the technology and the progress towards reliable applications and new discoveries[29].

## Methods

### Sample preparation and Genome Analyzer sequencing

The phiX174 Control Library used was prepared by Illumina (Cat. No CT-901-1001). Briefly, the double-stranded covalently closed circular form of the viral DNA was broken into 100–400 bp fragments by nebulization; the ends repaired with Klenow, T4 DNA polymerase and PNK; and a base *A *was added on the 3'ends. After ligation of the double-stranded genomic adapters the sample was gel-purified to isolate fragments with "inserts" of approximately 200 bp and amplified by 18 cycles of PCR (Illumina protocol "Preparing Samples for Sequencing Genomic DNA", Part # 11251892 Rev. A). The library is quality controlled by cloning an aliquot into a TOPO plasmid and capillary sequencing 5–10 clones.

DNA Colonies were prepared by using a "Standard Cluster Generation Kit" (Cat. No. FC-103-1001) and 35 cycles of isothermal amplification in the flow-cell on the "Illumina Cluster Station" using a pM dilution of the 10 nM library. After amplification, one of the strands is removed; the free 3'-ends are blocked by terminal transferase in presence of dideoxynucleotides; and the genomic sequencing primer hybridized. The flow-cell was transferred to the Genome Analyzer "classic" and sequencing was performed for 36 cycles using a "36 Cycle Sequencing Kit" (Cat. No FC-104-1003) with the version 2.0 of the scanning buffer.

### Sequencing of Human cells

The samples used for Additional file 3 came from the pooled DNA obtained by long-range PCR amplification[30] of a 30 kb region of chromosome 19 from 3 different individuals plus a 50 kb region of chromosome 3 from a fourth individual. Sequencing was performed as described above for the phiX174.

### Data analysis

All data analysis for this paper has been performed with the R statistical framework  and the Rolexa package. This package uses the *mclust *routines[20] as well as the *fork *package to run efficiently on multi-core architectures. Matching of short tags onto the genome have been performed with the *fetchGWI *tool[24] by first generating a comprehensive index of the phiX174 genome and matching each query with its index entry. We used *align0 *[25] to search for best matches from tags to the genome and estimate error rates (see Fig. 5A). When counting errors, an alignment of IUPAC code with one of its compatible bases was counted as correct match.

Raw data analysis (image analysis, initial base calling and fast-q scores) used the *Firecrest *image analysis module and the *Bustard *base-caller from the Illumina software suite (SolexaPipeline-0.2.2.6). No filtering or analysis with *Gerald *was performed.

### Preliminary data transformation

We model the measured intensities I(*α*, *n*, *x*) (*α *= *A*, *C*, *G*, *T *is the dye channel, *n *= 1, ..., *36 *is the cycle number and *x *denotes the colony coordinates) as the following combination of unbiased intensities *J*(*α*, *n*, *x*):

$$I(\alpha ,n,x)=\sum_{m=1,...,n}\sum_{\beta =A,C,G,T}M(\alpha ,\beta )J(\beta ,m,x)R(m,n),$$

where the 4 × 4 matrix *M *is a mixture matrix which is block diagonal and depends on the 4 parameters *ϕ*_*AC*_, *θ*_*AC*_, *ϕ*_*GT *_and *θ*_*GT*_:

$$M\left(\left\{A,C\right\},\left\{A,C\right\}\right)=\left(\begin{array}{cc} cos\theta_{AC} & \sin\theta_{AC}\\ \cos\varphi_{AC} & \sin\varphi_{AC} \end{array}\right),$$

and similarly for the *G*, *T *block, and the dephasing matrix *R *is a function of the parameter *q *and has a binomial structure:

$$R(m,n)=\{\begin{array}{c} 0\text{ if }m>n,\\ \left(\begin{array}{c} n\\ m \end{array}\right)q^{n-m}{\left(1-q\right)}^{m}\text{ if }m\leq n. \end{array}$$

The parameters *ϕ*_*AC*_, *θ*_*AC*_, *ϕ*_*GT*_, *θ*_*GT *_are determined by minimizing the following function:

*F*_*n*_(*θ*_*AC*_, *ϕ*_*AC*_, *θ*_*GT*_, *ϕ*_*GT*_) = cor(*M*^-1^*I *(*A*, *n*, •), *M*^-1 ^*I*(*C*, *n*, •))^2 ^+ cor(*M*^-1 ^*I*(*G*, *n*, •), *M*^-1^*I*(*T*, *n*, •))^2^,

which defines an intermediate intensity matrix *K *= *M*^-1 ^*I*. This is then introduced into the function

$$G(q)=\sum_{\alpha ,n}cor{\left(R^{-1}K(\alpha ,n,•),R^{-1}K(\alpha ,n+1,•)\right)}^{2},$$

which is minimized to determine *q*.

Lastly, we correct systematic bias in function of the cluster coordinate as follows: we fit a 2-dimensional lowess [18] as a function of *(x*, *y) *coordinates and then subtract the difference between that fit and the median intensity across all four channels, for each tile and cycle.

### Model-based clustering and data fitting

We used the *EEV *model of the *mclust *algorithm[20] to fit the Gaussian mixtures used to assign base probabilities in function of the four-dimensional intensity vector, similar as what was performed in [12]. This model assumes Gaussian mixtures with four covariance matrices of the same shape and volume but with varying orientation. We initialize the classification by attributing each colony to the nucleotide with the highest (corrected) intensity. Given that initial classification, an M step of the *mclust *algorithm is performed which estimates the maximum likelihood parameters given the class attributions, where the parameters to estimate are the global scale and shape parameters as well as the centers and orientations of each class (using the covariance parameterization described in [20]). This is then followed by an E step of the EM algorithm to estimate the conditional probabilities of each data point belonging to each class given the parameters estimates obtained previously. Full convergence of the EM algorithm is offered as an option but occasionally runs into spurious optima due to the effect of outliers (similarly to what was observed in [12]). Further details of the implementation can be found in the package documentation (see Availability section).

### Cutoffs for base calling and tag length

The Rolexa algorithms require two types of cutoffs, which can both be easily user-defined in the Rolexa package. In the analyses presented, the limits between the different IUPAC bases in the probability simplex (Figure 2A) were set to *HT(n) *= log_2_(*n*+0.5) with *n *= 1,2,3 (Figure 2B). Secondly the length-dependent cutoffs *IT(n) *were used to filter out uncertain bases by selecting the longest sub-tag *S *with total entropy smaller than *IT(n *= length *(S))*. In Figure 6 we used the following 6 choices: constants *IT*_*c*_*(n) *= *c *with the constant *c *set to 2, 4, 6, or 8, and two cutoffs increasing with the tag length: *IT*_Log _(*n*) = log_2 _(4 + (*n *- 1)/5) and *IT*_Exp _(*n*) = 2^(1+(*n*-1)/36)^. The latter two cutoffs interpolate between 2 and approximately 4 over the length of the sequence, but the first cutoff is concave (increases faster at the beginning) and the second is convex.

### Availability

We have developed an R package called Rolexa which is freely available from . It is distributed under the GPL license and uses the *mclust *package which is part of the R distribution.

## Authors' contributions

JR and AA implemented the method, JR and CI analyzed the data, JR and FN wrote the manuscript, FN and IX designed and supervised the study. LF provided insight and data and performed the experiments. All authors read and approved the final manuscript.

## Supplementary Material

Additional File 1 **Signal over noise decays with sequencing cycle number**. Histograms of the raw fluorescence intensities are shown for cycles 5, 15, 25, and 35. The separation between signal and noise is increasingly blurred and faster in the *A *and *G *channels than in the *C *and *T *channels. Red lines indicate a fit by a mixture of two Gaussians distributions with blue vertical bars indicating the mean and one standard deviation for the highest component of the mixture. Click here for file

Additional File 2 **Correction of positional bias**. **A**. Images show local averages of the fluorescence intensities across the area of a tile. The center of the tile is clearly brighter than the edges. **B**. After correction by lowess fit, the averages are visually more constant across the tile. Click here for file

Additional File 3 **Increased coverage of Rolexa data relative to Solexa data on a human sample**. A complete sequencing lane (330 tiles) was analyzed with Rolexa and Solexa pipelines. The X axis represents the number of nucleotides covered by the sequences of a tile with Rolexa base-calling and the Y axis represents the ratio with the corresponding Solexa base-calling with tags restricted to 25 bases or the full 36 bases length. Click here for file
