# Supplementary figures and images for: Probabilistic base calling of Solexa sequencing data
Source: BMC Bioinformatics. 2008 Oct 13;9:431. doi: 10.1186/1471-2105-9-431 (PMC2575221; doi:10.1186/1471-2105-9-431)

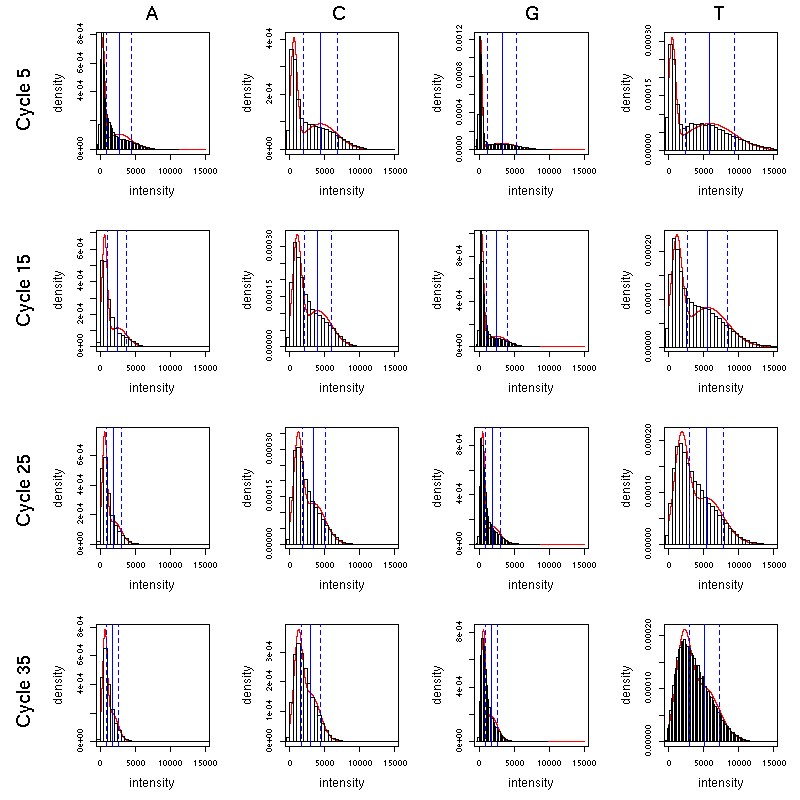

Supplement: Additional File 1 — Signal over noise decays with sequencing cycle number. Histograms of the raw fluorescence intensities are shown for cycles 5, 15, 25, and 35. The separation between signal and noise is increasingly blurred and faster in the A and G channels than in the C and T channels. Red lines indicate a fit by a mixture of two Gaussians distributions with blue vertical bars indicating the mean and one standard deviation for the highest component of the mixture. [file 1471-2105-9-431-S1.png]

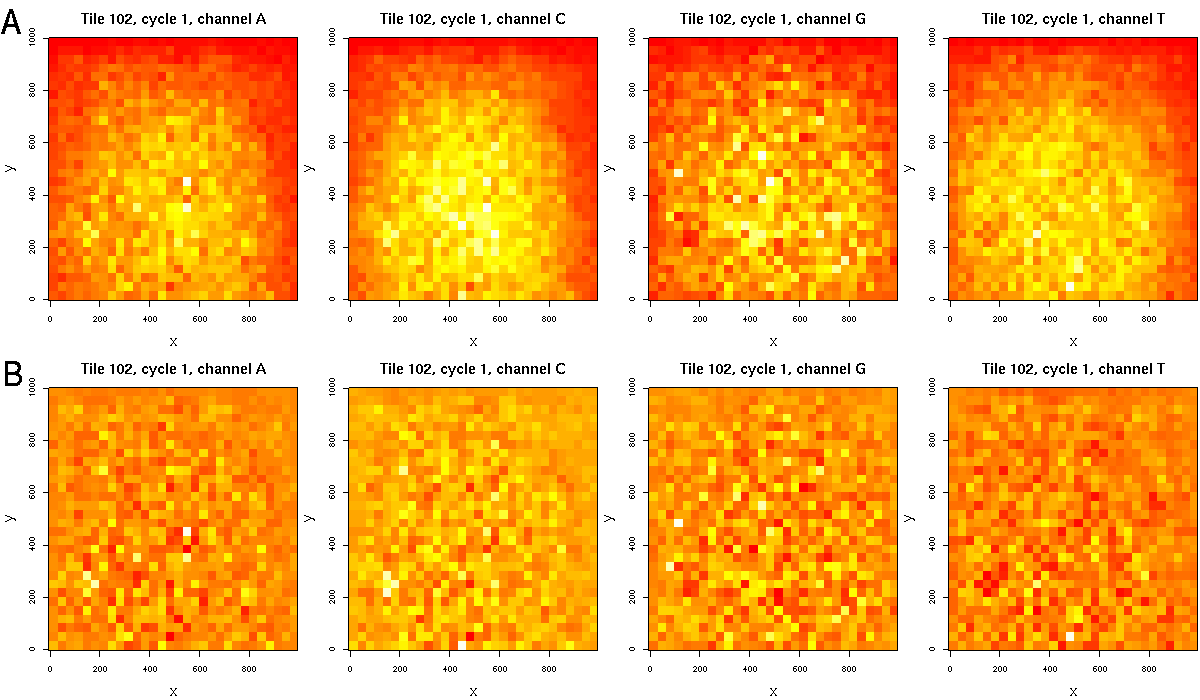

Supplement: Additional File 2 — Correction of positional bias. A. Images show local averages of the fluorescence intensities across the area of a tile. The center of the tile is clearly brighter than the edges. B. After correction by lowess fit, the averages are visually more constant across the tile. [file 1471-2105-9-431-S2.png]

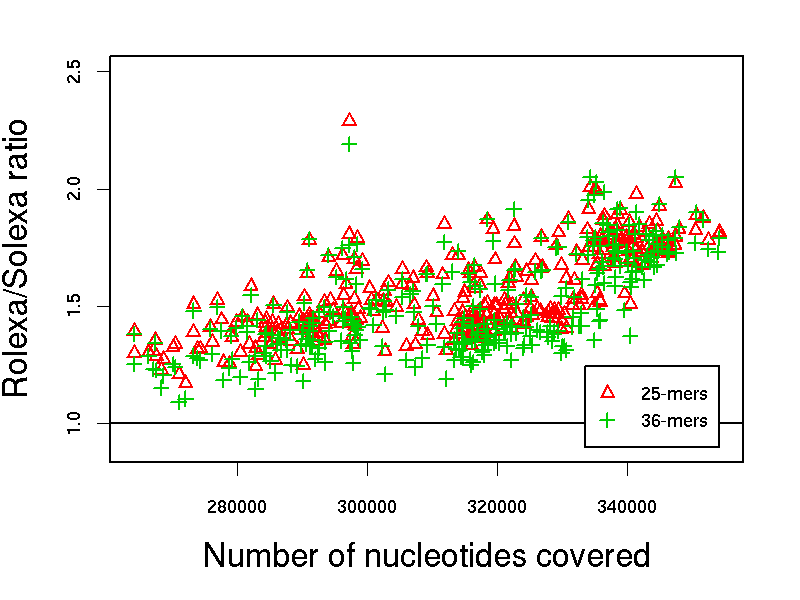

Supplement: Additional File 3 — Increased coverage of Rolexa data relative to Solexa data on a human sample. A complete sequencing lane (330 tiles) was analyzed with Rolexa and Solexa pipelines. The X axis represents the number of nucleotides covered by the sequences of a tile with Rolexa base-calling and the Y axis represents the ratio with the corresponding Solexa base-calling with tags restricted to 25 bases or the full 36 bases length. [file 1471-2105-9-431-S3.png]
